# Phosphorus monolayer doping (MLD) of silicon on insulator (SOI) substrates

**DOI:** 10.3762/bjnano.9.199

**Published:** 2018-08-06

**Authors:** Noel Kennedy, Ray Duffy, Luke Eaton, Dan O’Connell, Scott Monaghan, Shane Garvey, James Connolly, Chris Hatem, Justin D Holmes, Brenda Long

**Affiliations:** 1 School of Chemistry, University College Cork, Cork, Ireland; 2 Tyndall National Institute, Lee Maltings, Cork, Ireland; 3 Applied Materials, Lee Maltings, Cork, Ireland; 4 Applied Materials, Gloucester, Massachusetts, USA; 5 CRANN@AMBER, Trinity College Dublin, Dublin 2, Ireland

**Keywords:** CMOS, doping, monolayer, silicon, silicon on insulator (SOI)

## Abstract

This paper details the application of phosphorus monolayer doping of silicon on insulator substrates. There have been no previous publications dedicated to the topic of MLD on SOI, which allows for the impact of reduced substrate dimensions to be probed. The doping was done through functionalization of the substrates with chemically bound allyldiphenylphosphine dopant molecules. Following functionalization, the samples were capped and annealed to enable the diffusion of dopant atoms into the substrate and their activation. Electrical and material characterisation was carried out to determine the impact of MLD on surface quality and activation results produced by the process. MLD has proven to be highly applicable to SOI substrates producing doping levels in excess of 1 × 10^19^ cm^−3^ with minimal impact on surface quality. Hall effect data proved that reducing SOI dimensions from 66 to 13 nm lead to an increase in carrier concentration values due to the reduced volume available to the dopant for diffusion. Dopant trapping was found at both Si–SiO_2_ interfaces and will be problematic when attempting to reach doping levels achieved by rival techniques.

## Introduction

Aggressive device scaling in the sub-20 nm region has resulted in a number of techniques that were previously essential being deemed detrimental to current and future device production. Semiconductor substrates require doping to reduce their resistivity and enable their use in electronic devices such as metal-oxide semiconductor field-effect transistors (MOSFETs). Traditionally, ex situ doping was carried out using ion implantation, which suffers from several downsides when used on sub-10 nm devices and with three-dimensional architectures [1–2]. The main issues with ion implantation are that it introduces crystal damage that cannot be annealed out of these extremely small sub-10 nm devices, and that it is unable to conformally dope three-dimensional nanostructures due to the directionality of the technique. Ion implantation operators have devised several methods to counter these issues such as hot implantations but have shown only moderate success [3–4].

The introduction of crystal damage has major consequences when preparing devices for applications in the electronics industry such as CMOS. The short-channel effect (SCE) becomes more profound with reduced device dimensions and when combined with crystal damage leads to high leakage currents, which result in elevated power consumption. Therefore, it is essential for future device scaling that a means of damage-free, conformal doping is established, and this is where monolayer doping (MLD) appears to have potential to succeed.

**Figure 1:**
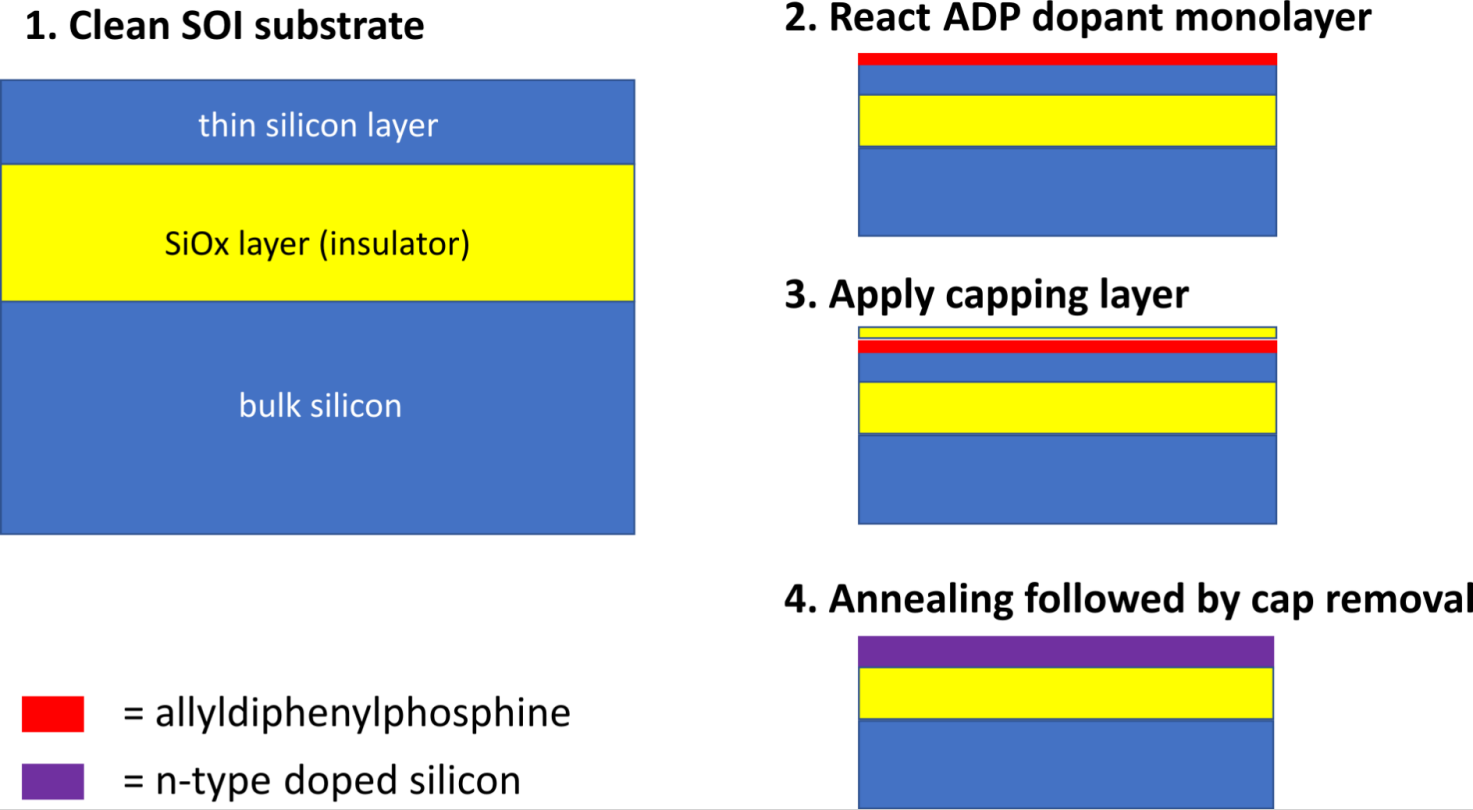
Schematic depicting MLD processing applied to silicon on insulator wafers. It shows monolayer formation (allyldiphenylphosphine dopant molecules) followed by capping and finally thermal annealing and cap removal to provide an n-type doped silicon layer.

MLD was pioneered by Javey and co-workers [5] in 2008 and has subsequently been used to dope multiple substrate types such as silicon [5–8], germanium [9–11] and others [12]. MLD involves the use of surface chemistry to provide a source of dopant atoms for diffusion into the substrate. Figure 1 shows a schematic version of the steps involved in a MLD process. The most commonly used reaction involves the hydrosilylation of an allyl-containing dopant molecule by a hydrogen-terminated silicon surface (produced using hydrofluoric acid). A capping layer is then applied to the sample followed by thermal treatment to promote diffusion of the dopant atoms into the silicon substrate while also providing enough energy to activate them in the crystal structure. By contrast, Ye et al. have recently proposed a monolayer contact doping (MLCD) process without the need for a capping layer [13].

This paper will examine the application of phosphorus MLD to silicon on insulator (SOI) substrates with nanoscale dimensions (sub-66 nm silicon layer). Bulk silicon transistors encounter difficulties when scaled below 20 nm due to SCE and significant leakage currents, which increase their power consumption. SOI and three-dimensional finFET structures are two means of device scaling that are currently being pursued by the electronics community. Planar, fully depleted SOI (FD-SOI) has been used to provide a more cost-effective scaling mechanism than FinFET alternatives. Although initial wafer cost is higher for SOI compared to bulk silicon, which is used in finFETs, the further masking and etching required for fin production is both complex and expensive. SOI allows for excellent electrostatic control of the channel without needing to dope this channel. Ultra-thin body SOI is also known to be high speed with low power consumption and low parasitic capacitance [14]. SOI doping has applications in a variety of fields including electronics, thermoelectrics and photovoltaics. MLD is capable of damage-free source/drain doping of planar SOI. There have been no previous publications of MLD on SOI substrates, which due to their confined dimensions, may provide an opportunity to limit dopant atom diffusion and therefore achieve active carrier concentrations greater than those that would be expected in bulk silicon.

## Results and Discussion

1 × 1 cm bulk p-type silicon and SOI samples were cut, and hydrogen-terminated using 2% hydrofluoric acid. The functionalization procedure was then carried out as outlined in the Experimental section. Allyldiphenylphosphine (ADP) was used as the dopant molecule in view of its commercially availability and relatively small size. ADP also minimizes the possibility of multilayer formation because it contains two unreactive phenyl functional groups.

**Figure 2:**
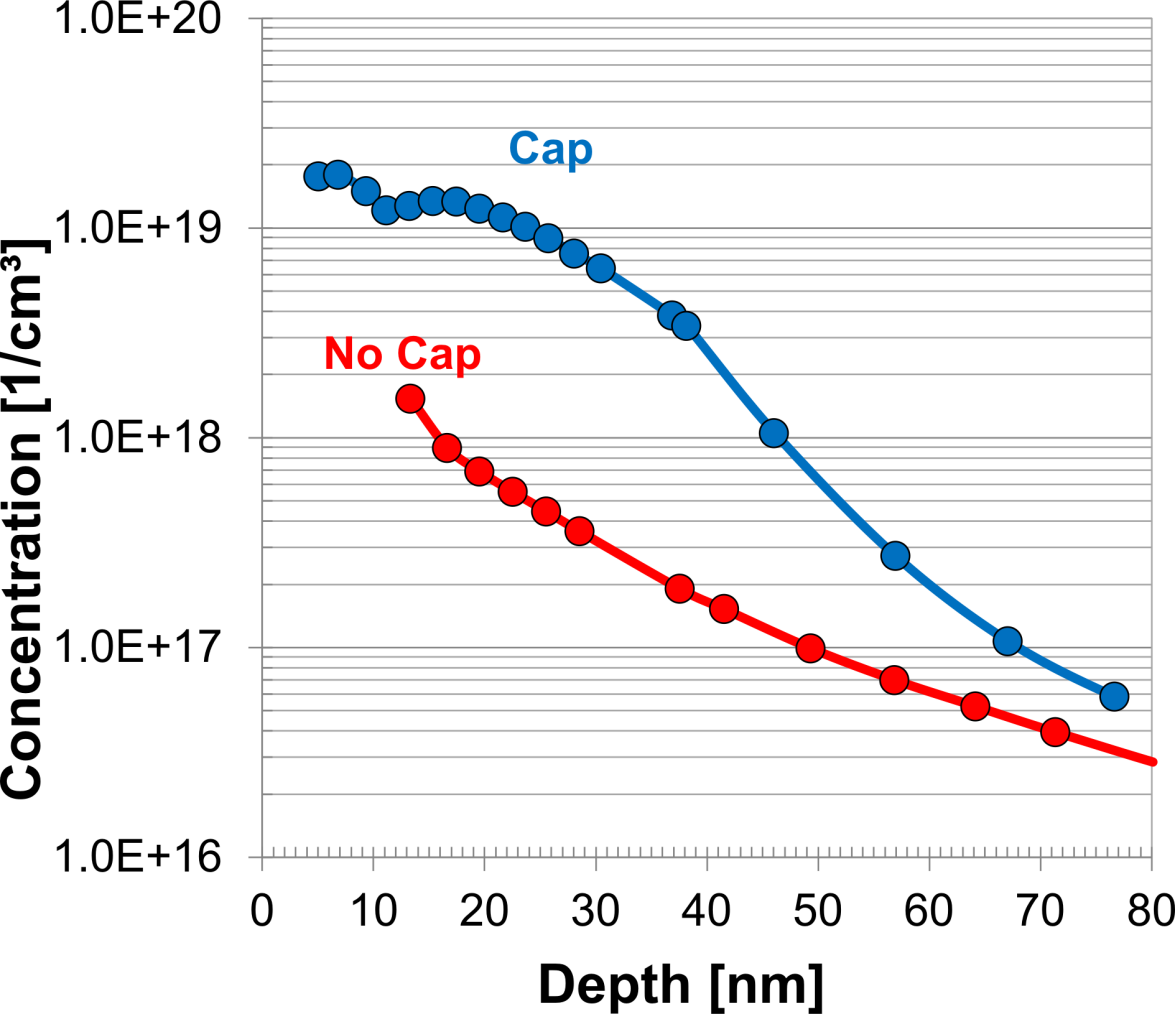
Electrochemical capacitance–voltage profile showing the impact of applying a SiO_2_ capping layer for the duration of the annealing process. Both samples were annealed at 1050 °C for 5 s (the inset shows the allyldiphenylphosphine dopant molecule).

Initial tests were carried out to determine whether a capping layer was necessary when carrying out phosphorus MLD. This was done using bulk silicon samples. Electrochemical capacitance–voltage (ECV) profiling is a technique that analyses the quantity of active dopant atoms present in a substrate as a function of the depth. Figure 2 shows that the application of a capping layer is necessary to achieve maximum dopant incorporation when carrying out P-MLD using ADP as the dopant molecule. SiO_2_ was chosen as capping material due to the poor diffusivity of P through SiO_2_, which would favour the preferential diffusion of P into the silicon substrate. Without the protection of a capping layer the dopant monolayer is essentially “burnt” off during high-temperature annealing. Cap removal was carried out using a standard buffered oxide etch.

**Figure 3:**
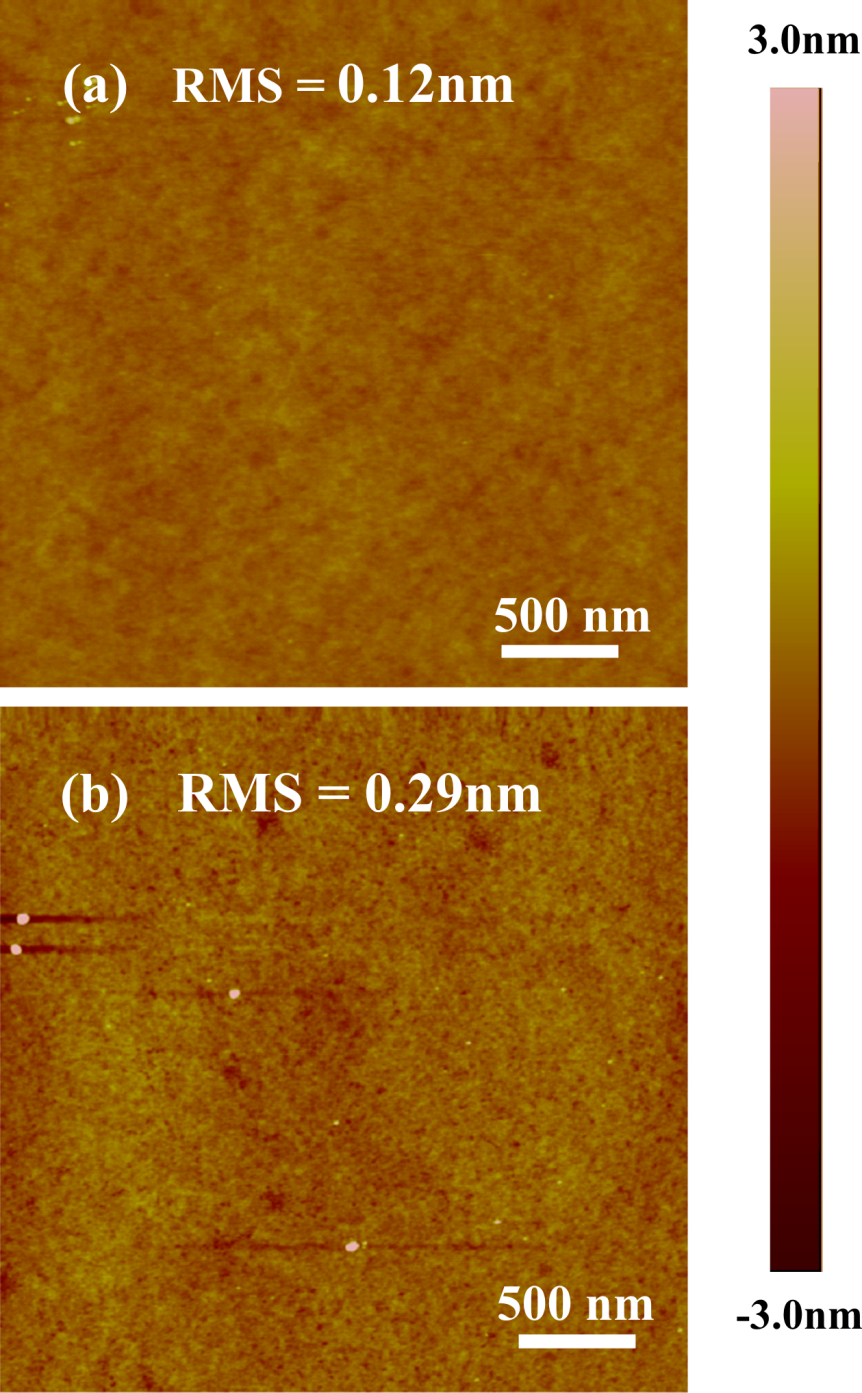
AFM images of (a) as received SOI (b) SOI after MLD processing.

Atomic force microscopy (AFM) was used to acquire high-resolution topographic images to evaluate the surface quality throughout MLD processing. Starting wafers were of good quality showing roughness values (RMS) below 0.2 nm (Figure 3). After MLD processing, the roughness values slightly increase to approximately 0.3 nm but this may be due to small oxide fragments on the surface, which remain from the cap removal process. Otherwise the surface quality remains relatively smooth. These values are important for both further analysis and industrial applications of MLD on SOI. The carrier-concentration analysis techniques ECV and Hall effect measurement both require high-quality surfaces and substrates to provide accurate data. Furthermore, from an industrial point of view it is important that surface quality remains good to ensure reproducibility over large sample quantities.

**Figure 4:**
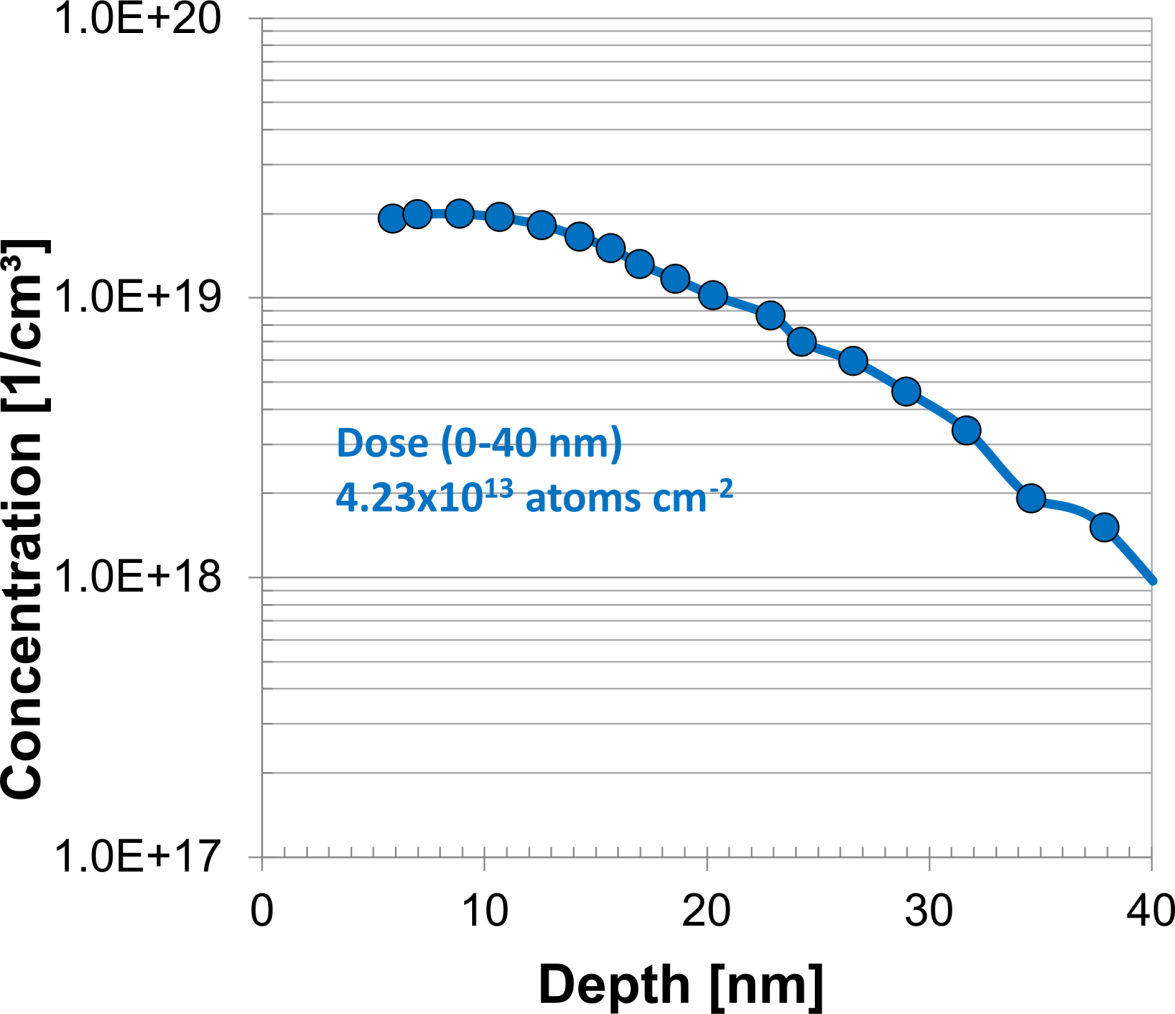
ECV plot of active carrier concentrations in a 66 nm SOI after MLD using a 50 nm sputtered SiO_2_ cap and annealing at 1050 °C for 5 s.

P-MLD processing was carried out on 66 nm SOI wafers through the methods outlined in the Experimental section. The active carrier concentration levels shown in Figure 4 approach 2 × 10^19^ cm^−3^, which correlate with the results seen during the initial capping test carried out on bulk substrates. This data shows that, as expected, MLD is applicable to SOI substrates. A comparison with 13 nm substrates will demonstrate the effect of confining the dopant diffusion.

**Figure 5:**
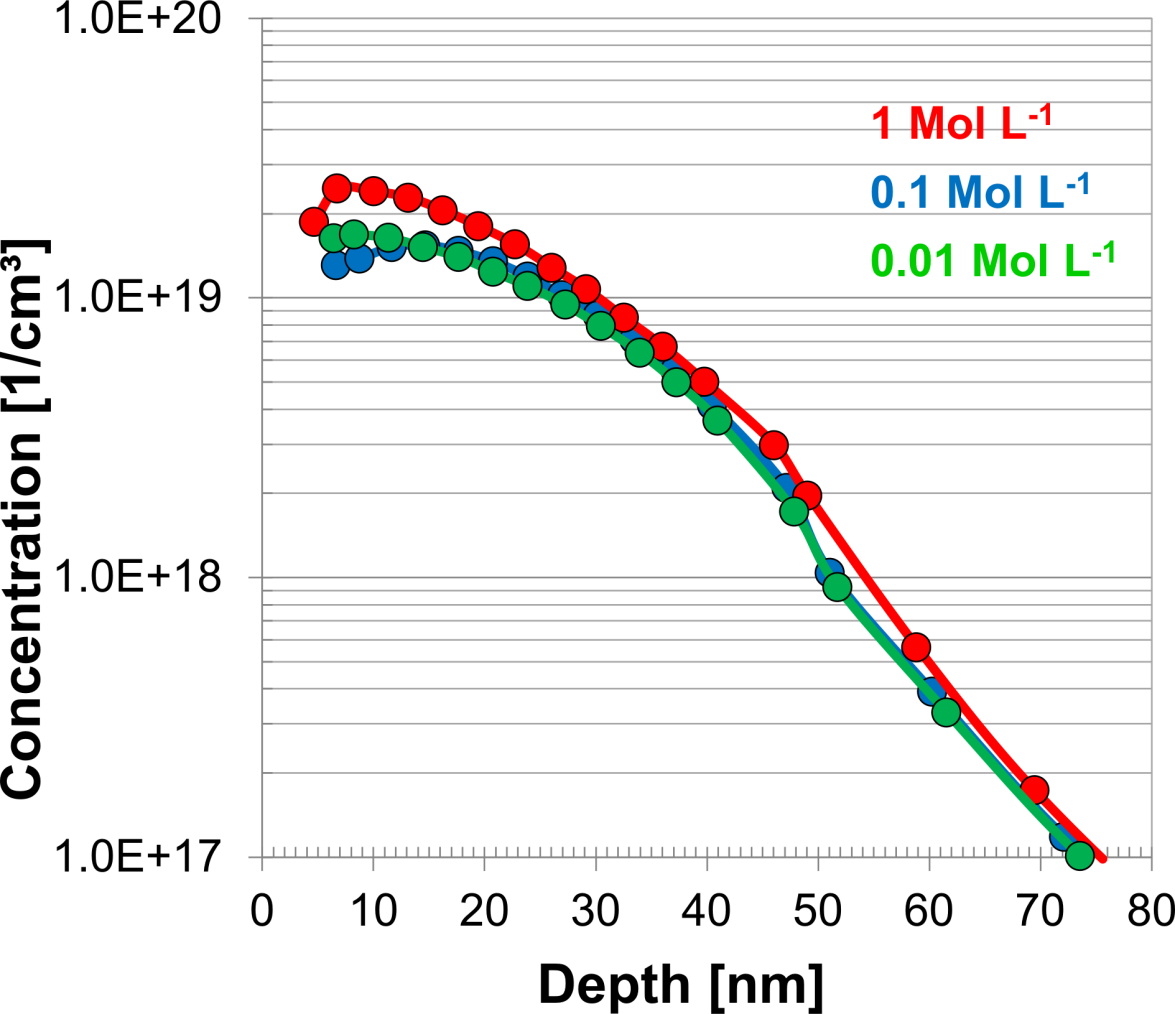
ECV plot of active carrier concentrations using bulk silicon samples to analyse the variation of the molecule concentration during functionalization. A 50 nm sputtered SiO_2_ cap and annealing at 1050 °C for 5 s was used for all samples.

It is also important to note that functionalization was carried out using a low concentration of ADP (0.1 M = 2% v/v). Even at these low levels it was found on bulk silicon substrates that ADP produced optimal active carrier concentration levels after processing with a functionalization of 3 h shown in Figure 5.

13 nm SOI samples were prepared and MLD-doped through the methods outlined in the Experimental section. ECV was not applicable to analyse active carrier concentrations present in these samples due to their inability to etch. When etching n-type doped semiconductors, ECV requires the application of a voltage to draw holes to the surface and enable the dissolution of the semiconductor into the electrolyte. Applying this voltage near the insulator layer becomes problematic and prevents etching and analysis in this region. Hall effect measurements were instead used, which required careful handling during wet-chemistry functionalization due to the precise dimensions needed for analysis. The Hall measurement system applies current and magnetic field and measures voltages and resistances. It then infers mobility and carrier properties from these measurements. The sheet resistivity (ρ_s_) is directly measured first by the four-point method, followed by the sheet Hall coefficient (sheet Hall resistance divided by magnetic field) as measured by Hall effect, *R*_HS_ = *V*_H_/(*I*·*B*), where *V*_H_ is the measured Hall voltage, *I* is the applied current and *B* is the applied magnetic field. Since ρ_s_ and *R*_HS_ are now directly measured and *R*_HS_ = ρ_s_·µ_H_, we can now infer the Hall mobility, µ_H_. The sheet carrier concentration (*n*_s_) is obtained from *R*_HS_ = *h*_f_/*n*_s_·*e*, where *e* is the electron charge. In dc mode, the carrier type is determined by the sign of the Hall voltage (negative = n-type, positive = p-type). In ac mode, the carrier type is determined by the phase of the Hall voltage (±180° = n-type; ±0° = p-type). Finally, applying a known or assumed thickness can convert these sheet properties to thickness-dependent properties.

A summary of the key data found with Hall effect analysis is shown in Table 1 with a more comprehensive data set available in Table S1 (Supporting Information File 1). The sheet carrier concentration (CC, dose) values, from ac mode, are virtually the same for both the 13 and 66 nm substrates. This is due to the overall dose available being limited by surface coverage of the ADP dopant molecule. Consistent dose values produced by MLD are desirable when compared with fluctuations seen using other techniques. However, the volume of the 13 nm samples is significantly less than that of the 66 nm sample, which leads to a higher carrier concentration (CC, *n*; concentration = dose/thickness). This is a very positive outcome. As a result of the increased carrier concentration the mobility drops, which is expected for silicon [15].

**Table 1:** Hall effect data of 66 nm and 13 nm MLD-doped SOI.

property	unit	66 nm sample	13 nm sample

mobility µ_H_	cm^2^·V^−1^·s^−1^	125.72	61.79
sheet CC	cm^−2^	2.3 × 10^13^	2.26 × 10^13^
CC, *n*	cm^−3^	3.49 × 10^18^	1.74 × 10^19^

### Dopant trapping

MLD is a surface-diffusion technique in which the dopant source is applied to the substrate surface and requires further thermal treatment to promote diffusion into the substrate and to electrically activate these dopant atoms. Although this process sounds trivial, there are numerous issues that can arise and prevent the movement of the dopant into the target area. In the case of silicon doping the most prominent issue is the silicon oxide formation at the surface. Phosphorus diffuses through silicon oxide significantly slower than through silicon [16–17]. Although it has been shown that hydrogen-terminated silicon re-oxidizes relatively slowly when stored at room temperature in air [3], the elevated temperatures required for MLD processing carried out in the liquid phase enhances this re-oxidation. Therefore, precautions are taken to ensure a minimal re-oxidation, i.e., solvents are thoroughly degassed, and processing is carried out in a N_2_ environment using a Schlenk line.

**Figure 6:**
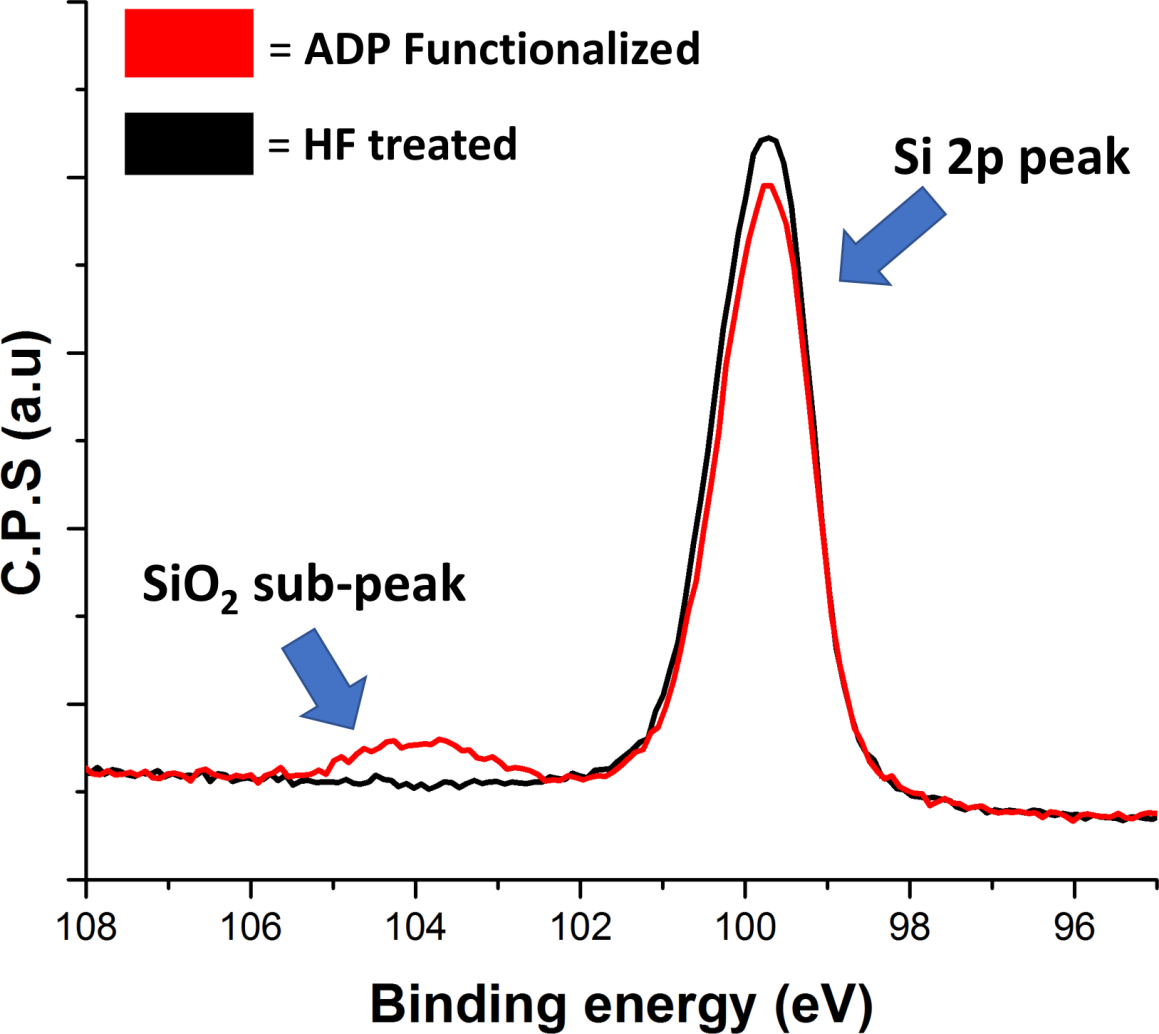
X-ray photoelectron spectroscopy (XPS) study showing that there is a degree of surface oxidation after functionalization procedure even when carried out under inert conditions.

XPS analysis of samples immediately after functionalization indicated that surface oxidation had taken place during this process despite the care taken to avoid oxidation. The Si 2p peak shown in Figure 6 has a sub-peak at approximately 104 eV, which is a result of the presence of SiO_2_. The presence of even this small amount of SiO_2_ has the ability to inhibit P diffusion into the Si substrate.

**Figure 7:**
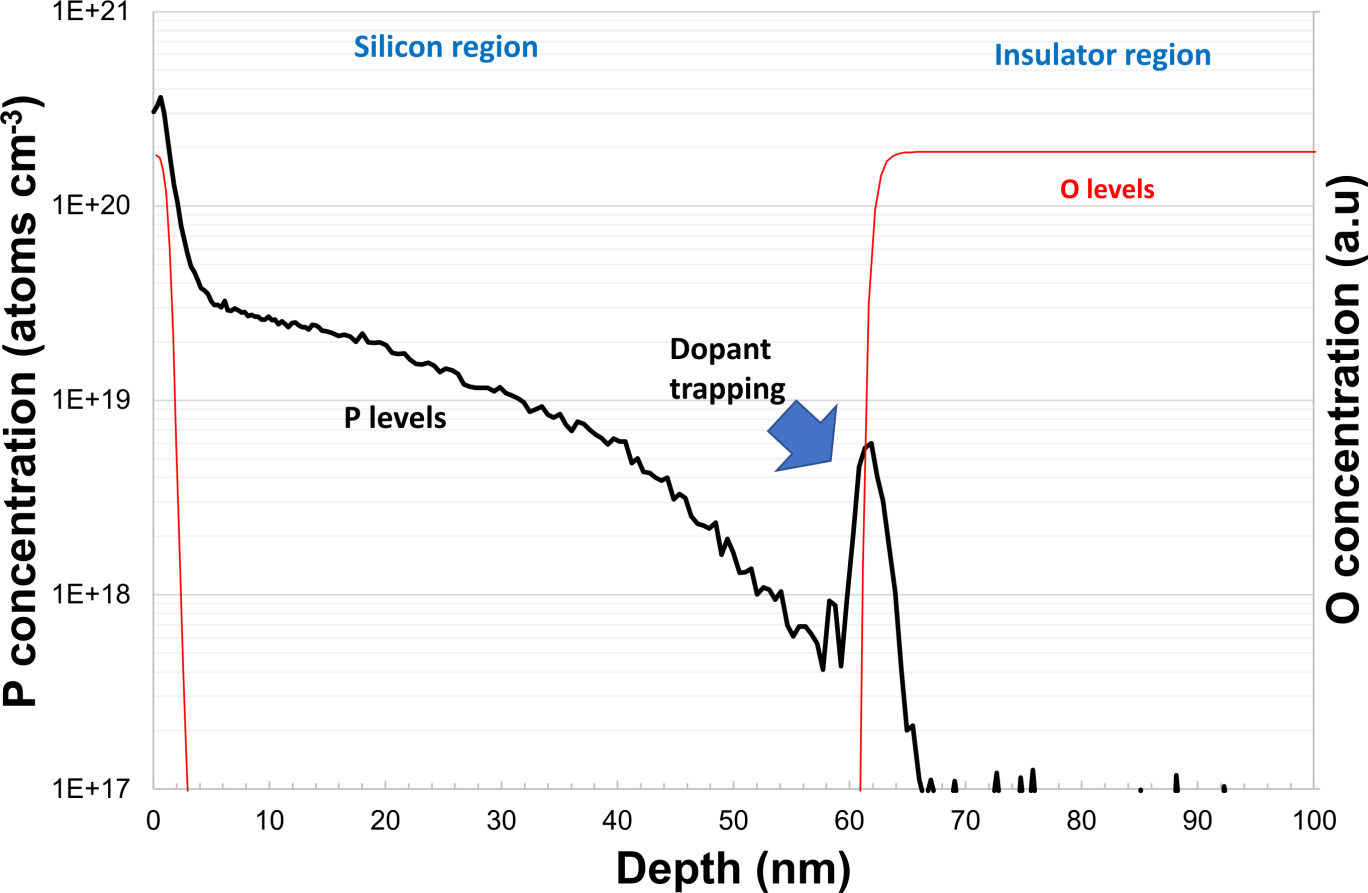
Secondary ion mass spectrometry analysis of a P-MLD-doped 66 nm silicon on insulator substrate. Blue line: P concentration, red line: O concentration.

MLD-doped 66 nm SOI was further examined using secondary ion mass spectrometry (SIMS) to attain a more detailed view of total dopant distribution in the substrate, which is complementary to previous measurements of active carrier concentrations through ECV. Data shown in Figure 7 correlates well with Hall effect and ECV measurements shown previously, with P concentration levels of 2 × 10^19^ cm^−3^ from 2 nm onwards, this shows that the majority of dopant atoms from this point are electrically active. The maximum levels found from SIMS were in the first 2 nm with values approaching 3 × 10^20^ cm^−3^. However, due to the inaccuracy of SIMS in this region it is difficult to assess these values. One possible reason for these elevated values may be dopant trapping by SiO_2_ during the annealing process. The surface oxidation found after functionalization (Figure 6) has the potential to inhibit diffusion into the substrate. Other research groups [7–8,18], working on P diffusion doping using a variety of techniques have also seen limitations at 2 × 10^19^ cm^−3^.

This was further examined by using longer annealing times of 10 and 100 s. Figure S1 (Supporting Information File 1) shows that this leads to an increased dose with maximum active carrier concentration levels remaining at 2 × 10^19^ cm^−3^. This leads us to believe that the presence of SiO_2_ near the sample surface may be inhibiting the in-diffusion of the P dopant atoms.

The final noteworthy aspect of this SIMS profile is the peak seen at the silicon–insulator interface. A spike in P concentration is seen showing that it may also be trapped at this point in the substrate. This spike could be explained by the slower diffusion of P in SiO_2_ compared to Si and a similar feature has been seen previously after ion implantation of SOI substrates [19]. A previous work by Mastromatteo et al. [20] examining P implantation of silicon nanocrystals embedded into SiO_2_ attributed a similar P peak to interface effects. It is unclear as to whether the silicon to insulator interface in these SOI substrates will behave in a manner similar to that of the silicon nanocrystals. In order to attain a more detailed understanding of this interface peak a more comprehensive study of this back interface would have to be undertaken.

## Conclusion

This study has demonstrated the first application of MLD to SOI substrates. Active carrier concentration levels attained in these substrates were consistently in the region of 2 × 10^19^ cm^−3^. Reducing the SOI dimensions did lead to an increase in carrier concentration (CC, *n*) found using Hall effect measurements. Further reducing the SOI dimensions into the sub-10 nm region will provide interesting knowledge around the application of P-MLD to ultra-thin SOI. Surface analysis showed that MLD processing caused minimal impact on sample surface quality and previous studies have also demonstrated the gentle nature of MLD on crystal quality. Dopant trapping at the Si–SiO_2_ interface appears to be a significant issue when applying MLD to SOI substrates. Considerable quantities of dopant atoms appear to be remaining in the surface region due to the presence of SiO_2_, which slows P diffusion. The use of more advanced techniques such as laser, flash lamp, and microwave annealing may solve this issue and allow for higher carrier concentration levels approaching the solid-solubility limits to be achieved in silicon.

## Experimental

### Substrate preparation

SOI samples were degreased through sonication in acetone for 120 seconds followed by a dip in 2-propanol and drying under a stream of nitrogen. Samples were then placed in a 2% HF solution for a period of 10 seconds to provide a hydrogen terminated surface. Following this HF treatment, the Si samples were dried under a stream of nitrogen and promptly placed under inert conditions in the Schlenk apparatus to prevent re-oxidation.

### Functionalization with ADP

All reaction steps were carried out under inert conditions on a Schlenk line apparatus. A solution of ADP in mesitylene (100 µL in 5 mL) was degassed using multiple freeze–pump–thaw cycles followed by transfer to the reaction flask containing the hydrogen-terminated silicon sample. This reaction flask was connected to a condenser that enabled reflux conditions during the 3 h heating period.

### Capping and annealing

A 50 nm SiO_2_ capping layer was sputtered on all samples prior to thermal treatments. Rapid thermal annealing was carried out allowing for temperatures greater than 1000 °C for time periods of less than 10 s, capable of producing ultra-shallow doping profiles. Capping layers were removed using a standard buffered-oxide etch (BOE). Optimal annealing conditions to provide high dose and active carrier concentrations while limiting the diffusion and junction depth were examined in Figure S1 and Figure S2 (Supporting Information File 1), which lead to the use of a 1050 °C annealing for a time period of 5 s for all applications to SOI.

### Characterisation

Atomic force microscopy was carried out in tapping mode at room temperature to analyse the surface quality throughout the MLD process. ECV profiling (CVP21 Profiler) was used to determine the active carrier concentrations in the samples after the doping process was completed. Ammonium hydrogen difluoride (0.1 M) was chosen as a suitable electrolyte/etchant as it can remove the native oxide layer without etching into the underlying substrate under neutral conditions. Controlled-voltage etching was carried out with step widths of 2–5 nm. Secondary ion mass spectrometry data was acquired on a Phi Adept 1010 using a 0.5–1.0 keV Cs^+^ bombardment with negative ion detection.

### X-ray photoelectron spectroscopy

XPS spectra were acquired on an Oxford Applied Research Escabase XPS system equipped with a CLASS VM 100 mm mean radius hemispherical electron energy analyser with a triple-channel detector arrangement in an analysis chamber with a base pressure of 5.0 × 10^−10^ mbar. Survey scans were acquired between 0 and 1400 eV with a step size of 0.7 eV, a dwell time of 0.3 s and a pass energy of 50 eV. Core-level scans were acquired at the applicable binding energy range with a step size of 0.1 eV, dwell time of 0.1 s and pass energy of 20 eV averaged over 10 scans. A non-monochromated Al Kα X-ray source at 200 W power was used for all scans. All spectra were acquired at a take-off angle of 90° with respect to the analyser axis and were charge-corrected with respect to the C 1s photoelectric line by rigidly shifting the binding energy scale to 284.8 eV. Data were processed using CasaXPS software where a Shirley background correction was employed.

### Hall effect measurements

Room temperature Hall effect measurements are performed using a controllable electromagnet in a LakeShore Model 8404 Hall effect measurement system (HMS) with dc and ac magnetic field capability in the range of ±1.7 T for dc, and of 1.2 T RMS (ac, 50/100 mHz), respectively. The ac magnetic field mode works in combination with a high-resolution lock-in amplifier that filters out all dc error components and uses phase analysis to remove ac error components. As a consequence, the ac results are generally more accurate that the dc results. Fitted with a high-resistance unit, the HMS can deal with many material systems that have low mobility, high resistivity and low carrier concentrations. As well as Hall effect measurements, the HMS also performs checks for ohmic behaviour and four-point resistivity measurements, and combines all-current/field-reversal techniques, optimisation methods and averaging between all geometries to remove most major error components and obtain an accurate Hall voltage assessed against the signal-to-noise (SNR) accuracy obtained [21]. For all samples assessed in this work, the coupon size is ca. 1 cm × 1 cm with four pressure probe metal contacts placed in the corners of the coupon, thus creating a van der Pauw structure [22]. The Hall factor (*h*_f_) is set to unity and the ac frequency is 100 mHz. We assume a uniform thickness with a uniform response across the material thickness. Moreover, the material is assumed to not have a dominant interlayer to be isolated electrically. If thickness-dependent properties are reported, we assume the thickness reported is correct.

## Supporting Information

Comprehensive Hall effect analysis data and ECV of annealing variation experiments on bulk silicon.

File 1 Additional experimental data.
