# Supplementary material for: Phosphorus monolayer doping (MLD) of silicon on insulator (SOI) substrates
Source: Beilstein J Nanotechnol. 2018 Aug 6;9:2106–13. doi: 10.3762/bjnano.9.199 (PMC6122086; doi:10.3762/bjnano.9.199)
Supplement: File 1 — Additional experimental data. [file Beilstein_J_Nanotechnol-09-2106-s001.pdf]

Supporting Information

for

Phosphorus monolayer doping (MLD) of silicon on insulator (SOI) substrates

Noel Kennedy*[1], Ray Duffy[2], Luke Eaton[1], Dan O'Connell[2], Scott Monaghan[2], Shane Garvey[2], James Connolly[3], Chris Hatem[4], Justin D. Holmes[1,5] and Brenda Long[1,2]

Address: [1]School of Chemistry, University College Cork, Cork, Ireland; [2]Tyndall National Institute, Lee Maltings, Cork, Ireland; [3]Applied Materials, Lee Maltings, Cork, Ireland; [4]Applied Materials, Gloucester, Massachusetts, USA and [5]CRANN@AMBER, Trinity College Dublin, Dublin 2, Ireland

Email: Noel Kennedy* - 111456882@umail.ucc.ie

* Corresponding author

Additional experimental data

Table S1: Comprehensive Hall effect data acquired from 66 nm and 13 nm P-MLD-doped SOI substrates.

Sample:		66nm		13nm (1of2)	
**Property**	**Units**	**Best DC**	**Best AC**	**Best DC**	**Best AC**
Thickness	nm	66	66	13	13
2P-4P current	A	50n-500n	50n-500n	50n-500n	50n-500n
2P Ohmic	none	0.997798	0.998587	1	1
2P Ravg	ohm	200k	175k	11.0k	10.8k
4P F-value	none	1	1	1	1
4P Ravg	ohm	480	475	1.0k	1.0k
Rc estimate	ohm	199.5k	174.5k	10.0k	9.8k
4P ρs	ohm/sq.	2.18k	2.16k	4.57k	4.46k
4P ρ	ohm.cm	14.37m	14.23m	5.94m	5.80m
d/dt change?	none	N/A	Yes	N/A	Some, ~2%
4P SNR est.	none	>~60:1	>~62:1	>~730k:1	>~744:1
Hall current	A	500n	500n	500n	500n
Hall B-field	T|T rms	1.7	1.24	1.7	1.24
Scan speed	none	N/A	Medium	N/A	Medium
Gain/Sense	dB/µV	N/A	0/500	N/A	0/500
Hall voltage	µV	-25.03	16.92	-25.93	17.21
C. Type/%	none	N/100	N/100	N/100	N/100
Geo error?	none	No	No	No	No
Mobility µH	cm2/(V.s)	135.24	125.72	66.82	61.79
Sheet CC, ns	1/cm2	2.12E+13	2.30E+13	2.05E+13	2.26E+13
CC, n	1/cm3	3.21E+18	3.49E+18	1.57E+19	1.74E+19
Rhs	cm2/C	2.94E+05	2.71E+05	3.05E+05	2.76E+05
Rh	cm3/C	1.94	1.79	0.397	0.358
Hall SNR est.	none	>~4:1	>~7:1	>~52:1	>~30:1
Phase	Degrees	N/A	-179.8	N/A	179.9

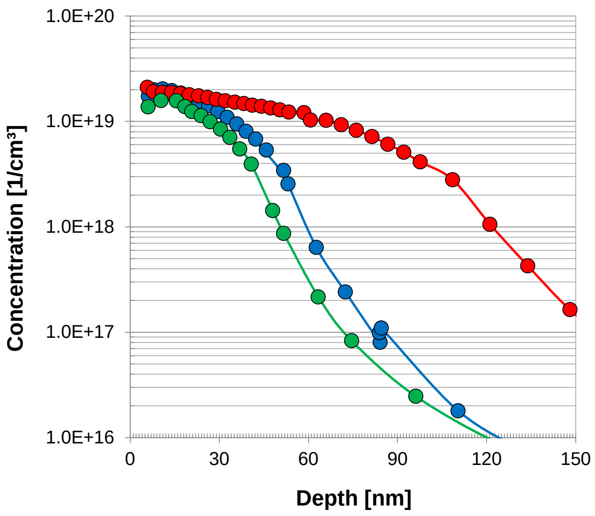

Figure S1: ECV profiles of the P-MLD on bulk silicon annealing time variation experiment. Samples were capped with 50 nm sputtered SiO_2 and annealed at 1050 °C for time periods of 5 s (green), 10 s (blue), and 100 s (red). The incorporated dose value after the 100 s annealing is 1.28×10^{14} cm^{-2}, which is close to what would be expected from a monolayer of allyldiphenylphosphine dopant molecules.

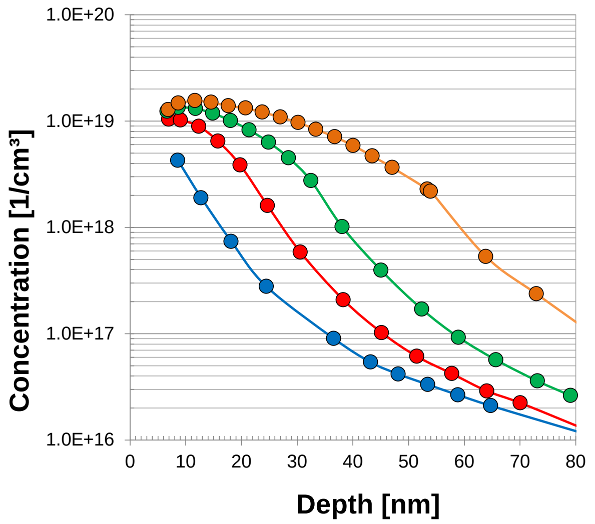

Figure S2: ECV profiles of P-MLD on bulk silicon annealing temperature variation experiment. Samples were capped with 50 nm sputtered SiO_2 and annealed at 950 °C (blue), 1000 °C (red), 1050 °C (green) and 1100 °C (orange) for a time period of 5 s.
